# Supplementary material for: Content-rich biological network constructed by mining PubMed abstracts
Source: BMC Bioinformatics. 2004 Oct 8;5:147. doi: 10.1186/1471-2105-5-147 (PMC528731; doi:10.1186/1471-2105-5-147)
Supplement: Additional File 2 — The original results of the above study (non-essential files are deleted to keep the file size under the limit set by BMC bioinformatics). [file 1471-2105-5-147-S2.bz2 › chilibotAdditionalFile2/dip10/327ID9546397E3626/html/TFRC_TF.html]

 


 **TFRC** and **TF** 
  
Found 3207 abstracts in PubMed, retrieved 10.  
 

 What does Google say? 
 PDF only 
| .edu only 

---

**Interactive relationship** (e.g. stimulation, inhibition, etc)

**Non-interactive relationship** (e.g. studied together, co-existance, homology, etc.)

- We further demonstrate that HOCl antagonizes the H2O2 mediated increase in the levels of transferrin  [ **TF** ]  receptor  [ **TFRC** ] , which is a downstream target of IRP1.  Ref: 12888561 J Biol ChemJ Biol Chem,
- One is the GFP high fraction containing mainly CFU E and proerythroblasts, which coexpress transferrin  [ **TF** ]  receptor  [ **TFRC** ] , while the other is the GFP low transferrin  [ **TF** ]  receptor  [ **TFRC** ]  negative fraction containing BFU E.  Ref: 12893747 BloodBlood,
- EXPLY 37 R had no effect on T lymphocyte activation, measured as proliferation as well as expression of early and late cell surface antigens CD69, CD25 IL 2R alpha and CD71  [ **TFRC** ]  transferrin  [ **TF** ]  receptor  [ **TFRC** ]  at the cell membrane.  Ref: 12890427 Int Immunopharmacol, 2003
- When fused to unpolarized CD4 or dendritic transferrin  [ **TF** ]  receptor  [ **TFRC** ] , T1 promoted their axonal surface expression.  Ref: 12893943 Science, 2003
- The geometric mean concentration of soluble transferrin  [ **TF** ]  receptor  [ **TFRC** ]  was lower in the exposed subjects than in the controls 2.2 vs 2.6 mg l 1.  Ref: 12884407 J Appl ToxicolJ Appl Toxicol, 2003
- RESULTS There were no significant differences in hemoglobin, serum transferrin  [ **TF** ]  receptor  [ **TFRC** ] , copper, or zinc among groups.  Ref: 12885712 Am J Clin Nutr, 2003
- Concentrations of hemoglobin, serum ferritin SF, and serum transferrin  [ **TF** ]  receptor  [ **TFRC** ]  TfR  [ **TFRC** ]  were measured at baseline and after 3 and 6 mo.  Ref: 12885710 Am J Clin Nutr, 2003
- An association was found between the concentration of serum soluble transferrin  [ **TF** ]  receptor  [ **TFRC** ]  and the concentration of Mn in whole blood Pearson s r = 0.48.  Ref: 12884407 J Appl ToxicolJ Appl Toxicol, 2003
- The regimen outlined in this brief paper focuses on therapeutically utilizing iron and iron compounds in transferrin  [ **TF** ]  receptor  [ **TFRC** ]  rich tumor cells in ways that inhibit and lyse same.  Ref: 12888304 Med Hypotheses, 2003
- There were no final differences between groups in hemoglobin, plasma ferritin, or transferrin  [ **TF** ]  receptor  [ **TFRC** ]  concentrations or in the ratio of transferrin  [ **TF** ]  receptors to plasma ferritin after control for initial concentrations.  Ref: 12885707 Am J Clin Nutr, 2003
